# Appropriate use of psychotropics in residential care facilities and important collaboration with clinical pharmacists

**DOI:** 10.1192/j.eurpsy.2026.10278

**Published:** 2026-07-31

**Authors:** D. Fialova, NETPHARM project WP4 group

**Affiliations:** Department of Clinical and Social Pharmacy, Charles University, Faculty of Pharmacy, Hradec Králové, Czech Republic

## Abstract

**Abstract:**

In 2023, the new project NETPHARM was granted in the Czech Republic, developing during the scientific works on WP4 „Geriatric clinical pharmacy and new technologies for the individualization of drug schemes in older patients” international research collaboration in highly individualized geriatric pharmacotherapy among clinical pharmacists, geriatricians and other medical specialities, including psychiatrists.

This project builds upon previous findings of the EU projects describing problems of irrational geriatric pharmacotherapy and importance of the implementation of clinical pharmacy services in different settings of care, e.g. the EUROAGEISM H2020 and I-CARE4OLD H2020 projects, EU COST Action IS1402 ESR7 program, INOMED, EU SHELTER 7th FP EC project and the START/MED/093 project. Using results of secondary analyses from available EU datasets utilized in the NETPHARM research, this lecture describes the situation in inappropriate geriatric prescribing of psychotropics in Central and Eastern Europe.

Results from regionally different nursing home facilities in 4 Central and Eastern EU countries - the Czech Republic (CZ), Bulgaria (BUL), Croatia (CRO), Slovakia (SLO), where in total 876 NH residents were assessed in 2020-2022 by the interRAI-LTCF international tool for comprehensive geriatric assessment (CGA), confirm the high rates of polypharmacy (49.6%, 5+ meds), hyperpolypharmacy (25.7%, 10+, meds), frequent use of psychotropic multicombinations (3+ psychotropics in 36.7% older patients) and overprescribing of benzodiazepines (BZDs), in some countries with the prevalence up to 30 % (CRO, SLO). BZDs were usually prescribed for 5+ years, in non-geriatric dosing (28%) and in risky drug multicombinations (52%). The odds of being prescribed hyperpolypharmacy was significantly higher in patients with moderate to severe frailty (CFS ≥5; proportional OR = 4.3), signalling the need for deprescribing initiatives and for optimizing pharmacotherapy in this multimorbid cohort of older patients.

This lecture documents core problems of irrational use of psychotropics in older adults in nursing homes in Central and Eastern Europe and increases awareness to important role of clinical pharmacists in multidisciplinary clinical teams in optimizing pharmacotherapy in older NH residents.

*Grant support: NETPHARM project CZ.02.01.01/00/22_008/0004607, co-financed by the European Union, Cooperatio research program of the Faculty of Pharmacy, Charles University (Research Unit KSKF-1, Chair: Assoc. Prof. Fialová) and SVV260665.*

**Disclosure of Interest:**

None Declared

